# A case study assessing energy-exergy-economic (3E) performance in solar air heaters with different winglet geometries and air flow rates

**DOI:** 10.1038/s41598-026-38467-x

**Published:** 2026-02-07

**Authors:** Vijayakumar Rajendran, Wesley Jeevadason Aruldoss, Vinoth Kumar Selvaraj, Jeyanthi Subramanian, Beno Wincy Winsly, Demoz Lisanework

**Affiliations:** 1 Centre for Smart Energy Systems, Chennai Institute of Technology, Chennai, Tamil Nadu India; 2 School of Mechanical Engineering, Vellore Institute of Technology, Chennai, Tamil Nadu India; 3 Department of Electromechanical Engineering, Faculty of Manufacturing, Institute of Technology, Hawassa University, Hawassa, Ethiopia

**Keywords:** Energy performance, Solar air heater, 3E analysis, Absorber plate design, Thermo-hydraulic efficiency, Heat transfer, Energy science and technology, Engineering

## Abstract

Solar air heater (SAH) research focuses on modifying absorber plates to enhance heat transfer through artificial roughness, fixing, and other cost-effective techniques. The objective of this investigation is to compare the energy, environmental, and economic (3E) performance of SAH with inclined triangular winglet (IWSAH) and sinusoidal winglet (ISWSAH) under diverse air flow rates (0.01 kg/s, 0.02 kg/s, and 0.03 kg/s). The results indicate that both winglet configurations exhibit improved performance, with IWSAH being considered the best. The output air temperature reaches a maximum of 82.7 °C at 0.01 kg/s flow rate in IWSAH and decreases as the airflow rate increases. With 1.12 times higher heat transfer coefficient, the average thermal efficiency of IWSAH reached 73.1% compared to 68.8% for ISWSAH. IWSAH achieves a net energy increase of approximately 4–6% more than ISWSAH. ISWSAH shows the maximum average heat loss when the airflow rate is at its minimum of 0.01 kg/s. A significant 52.3% gain in thermo-hydraulic efficiency occurs when the airflow rate is raised from 0.01 kg/s to 0.03 kg/s. This improvement is primarily due to the blower’s reduced power consumption in the IWSAH system, which was previously at 25.7%. IWSAH outperforms ISWSAH in both economic and environmental assessments. The economic and environmental analysis shows that the IWSAH system outperforms the ISWSAH system with a shorter energy payback time (1.33 vs. 1.62 years), higher energy production factor (2.96 vs. 2.71), better life cycle conversion efficiency (43.6% vs. 39.1%), and reduced emissions of CO2 (2147.2 vs. 2201.3 kg), NO (16.3 vs. 16.6 kg), and SO2 (6.7 vs. 6.9 kg), alongside lower annualized costs (₹3195.7 vs. ₹3268.5), demonstrating its superior economic and environmental viability.

## Introduction

 Solar air heaters (SAHs) consist of a chamber, a top glass cover, a heat-transfer medium (often air), a blower, and flow control mechanisms to distribute heated air as required. They play a crucial role in advancing sustainable energy systems in building applications by utilizing renewable energy, contributing to carbon reduction, and supporting the transition to cleaner energy sources. However, conventional SAHs often suffer from low thermal efficiency, primarily due to limited heat transfer capacity. Improving the heat transfer coefficient is therefore essential for achieving optimal performance. To enhance the efficiency of SAHs in building applications, various design modifications—such as adding fins, baffles, or channels—have been explored to increase turbulence and improve airflow across the absorber plate. These strategies have been widely investigated to address the thermal limitations of SAHs and boost their effectiveness in delivering sustainable heating solutions for buildings^1–3^.

An integrated arrangement of inclined and transverse ribs is investigated in an SAH with a roughness height of ribs as 0.030. The Re and roughness pitch are taken as variable and are varied between 2000 and 14,000 and 3–8, respectively. The outcomes portray that the SAH efficiency is greatly improved with the roughness. Among all the possibilities looked at, the design with a roughness pitch of eight had the maximum efficiency^4^. A SAH was evaluated for its performance using an equilateral triangular section that had a significant rib roughness. It revealed that the thermo-hydraulic performance of the rib arrangement ranged between 1.36 and 2.11^5^. An evaluation is conducted to determine the effectiveness of different rib orientations, including inclined, transverse, and V-shaped ribs, for SAHs. The research findings point out that inclined ribs have performed better than transverse ribs in thermo-hydraulic performance^6^.

A study examined improving the heat transfer in SAH by using a vortex generator with winglets. The results of this research show that as the attack angle is raised from 30° to 60°, the Nusselt number and its accompanying magnitudes increase continuously. The Nusselt number has reached a maximum at the angle of attack at 60°^7^. Different combinations of rectangular and triangular airflow tubes were positioned above and below the absorber plate as part of a new arrangement used in an experiment. The SAH’s performance was improved in all configurations^8^. A comparative analysis was conducted between a conventional SAH, and one attached with rectangular flow tubes. With respective values of 22.4% and 18.1%, the findings show a notable rise in thermal and effective efficiencies^9^. Six configurations with baffles such as transverse, inclined transverse, dimple, sine wave, and arc over the collector plate were used to examine the efficiency of an SAH. The investigation demonstrated that the sine wave baffle functioned better than any other design examined due to the more turbulence created by it^10^. The absorber plate in a SAH with flapped and punched delta-winglets was used separately to examine the thermo-hydraulic performance. The test findings revealed that the flapped and punched delta-winglet designs performed differently, by a factor of 2.16^11^. A new analysis was carried out to determine the energy and exergetic efficiency of a SAH that was fitted with winglet-type roughness components. The outcomes revealed that the thermal, effective, and energy efficiencies were much higher than those of a smooth plate design; the corresponding values were 2.12, 1.99, and 2.03 times higher^12^. Experiments on the effectiveness of three different configurations the arc, straight angle, and arc with rectangular holes show that the spiral SAH with rectangular holes performed better at gathering energy than the other two designs. Its efficiency is 13.19% higher than that of the arc spiral SAH with rectangular holes, 10.14% higher than that of the right angle spiral SAH, and 9.38% higher than that of both^13^. An investigation on the effect of attaching staggered cuboid baffles over the thermal performance of SAH is conducted for Re between 5080 and 10,160. The thermo-hydraulic characteristics was found improved and was ranged between 2.80 and 3.43^14^. A study on SAH performance improvement indicated that in comparison to a smooth duct, a hybrid staggered rib structure greatly enhanced the Nusselt number and friction factor by 3.16 and 2.57 times, respectively. The Nusselt number of broken arc rib designs is 1.22 times higher than the smooth duct. The thermo-hydraulic performance of the suggested arrangement was 2.3, an exceptional result^15^.

Twelve wavy roughness components with pitches (p) between 10 and 25 mm and rib heights (e) between 0.7 and 1.4 mm were investigated in a SAH system. The results of the study demonstrate a notable improvement in thermo-hydraulic performance, particularly when Re values in the range of 3800–18,000 are employed. Interestingly, it was shown that 1.96 was the optimal thermo-hydraulic performance value for a Re of 12 in this case. Ribs with a height and pitch of 0.7 mm and 15 mm respectively fit this optimal value^16^. The characteristics of fluid flow inside an SAH were examined using a new sinusoidal rib construction. At a Re of 15,000, which falls within the range of Re investigated, an amazing 69% thermal efficiency was achieved with this innovative rib design^17^. Analysis was conducted on a return flow SAH with a V-shaped roughness that was placed on both sides of the collector plate, with an airflow rate of 0.02 kg/s. The study indicated that the thermo-hydraulic performance is higher in the SAH when the baffles are fixed on both sides of the plate than the baffles above or below the absorber plate^18^. The thermal and frictional properties of a solid-liquid interaction with ribs formed like a sawtooth wave were analyzed using mathematical methods. The design incorporating saw-tooth wave ribs and ribbed absorber exhibited a noteworthy enhancement in thermo-hydraulic performance due to a substantial 53.3% rise in the Re^19^. A study examined the effectiveness of heat transfer for SAHs and found that the power, thermal, and overall efficiencies were, 12.9%, 56.7%, and 69.6% respectively^20^. Research was conducted to solve the shortcomings in the operation of an air heater. This was achieved by adding a PCM storage bed and adding PCM-filled baffles. The daily effective efficiency of the SAH with PCM-filled baffles was approximately 52% higher than that of an SAH equipped with a PCM storage bed and 57.9% over the SAH lacking PCM^21^. The thermal characteristics and heat transfer of a double-pass SAH were investigated in a study that used rectangular perforated blocks with transverse semicircular tubes. Results indicate that the thermo-hydraulic parameter reaches its maximum value of 2.51 at the Re of 3000^22^. A study used three different baffle designs to calculate the efficiency of a SAH. The findings show a significant improvement in Type III in all the power, energy, and exergy efficiencies. The increase in rates ranged between 2.4 and 4.3%, 12.9–20.0%, and 5.6–8.9%, respectively. According to the comprehensive study conducted, this type demonstrated the highest efficiency and the minimum amount of pressure loss, making it the most favorable option^23^. The thermal efficiency of spiral flow SAHs ranged from 40% to 59%, with the highest values seen at baffle heights of 110 mm and 130 mm. The highest thermal performance was seen for these particular geometries at an airflow rate of 0.0075 kg/s^24^.

### Research gap/problem statement

Conventional SAH are limited by low thermal efficiency due to inadequate heat transfer and high frictional losses, restricting their practical applicability in sustainable building systems. Although numerous studies have employed roughness elements, ribs, and baffles to enhance performance, most investigations focus predominantly on thermal analysis while overlooking the integrated impacts of energy, exergy, and economic parameters. Furthermore, limited research has explored the comparative performance of advanced winglet-based geometries—particularly inclined triangular and sinusoidal configurations—under realistic operating conditions. The following research gaps are identified to highlight the limitations in existing studies and to establish the need for a comprehensive evaluation of solar air heater performance under realistic operating and climatic conditions.

- Comparative evaluation of winglet-based geometries, particularly inclined triangular and sinusoidal winglet configurations, under similar operating conditions and varying mass flow rates, has not been thoroughly investigated.
- Previous designs mainly aimed to enhance local heat transfer but did not sufficiently address pressure drop effects or blower power consumption, which directly influence overall system economics.
- Few studies have incorporated environmental impact assessments or life-cycle economic evaluations, despite the importance of sustainable buildings and renewable heating applications.
- The lack of real-time empirical validation in tropical climatic conditions, such as in South India, limits the practical applicability of the results.

### Novelty and objective

The novelty and objectives of this research stem from addressing the identified gaps through an integrated experimental and analytical approach. The objectives below are formed to address the aforementioned research gaps.

- To experimentally evaluate Solar Air Hater under the influence of Inclined Triangular Winglet and Inclined Sinusoidal Winglet.
- To comparatively analyze the effect of geometry and flow dynamics on the Energy-Exergy-Economics (3E) framework of the Solar Air Heater.
- To evaluate the thermo-hydraulic efficiency of the solar air heater by quantifying the combined effects of heat transfer enhancement and pressure drop, and to assess its environmental impact through the estimation of CO₂, SO₂, and NOₓ mitigation potential achieved under actual operating conditions.

Compared to previous studies, which focused largely on thermal performance, the present research integrates energy, environmental, and economic (3E) analyses to enable a well-rounded performance evaluation for solar air heaters. This experimental study aims to conduct an assessment of SAHs fitted with inclined triangular winglets and those with inclined sinusoidal winglets at different airflow rates. Thermal efficiency, heat transfer capability, and thermo-hydraulic performance are compared for these two configurations regularly. To the best of the authors’ knowledge, the present study is the first experimental effort presenting a direct comparative assessment of the SAHs fitted with inclined triangular and inclined sinusoidal winglets tested under similar outdoor operating conditions within an integrated 3E frame, as summarized in Table 1.

**Table 1:** Comparison novelty of the proposed system with existing research.

Aspect	Existing research focus	Present study novelty
Analytical scope	Primarily focused on thermal performance enhancement using artificial roughness, ribs, or baffled geometries.	Integrates energy–exergy–economic (3E) performance evaluation within a single experimental framework, linking thermal to sustainability metrics.
Geometric configuration	Studied conventional shapes such as transverse, V-shaped, arc, and sine ribs without direct comparison among different winglet profiles.	Introduces a comparative study of two advanced winglet designs—inclined triangular (IWSAH) and sinusoidal (ISWSAH)—under controlled conditions.
Performance indicators	Emphasized heat transfer improvement or Nusselt number correlations; rarely included penalty factors like pressure drop or blower energy use.	Quantifies thermo-hydraulic efficiency accounting for both thermal enhancement and flow resistive losses, providing realistic efficiency indices.
Economic and environmental integration	Economic analysis, when included, was often limited to basic cost estimation without environmental linkage.	Incorporates economic and environmental assessments, including payback period, operating cost, and CO₂ mitigation, offering a sustainability-oriented perspective.

## Methodology and experimental setup

### Description of setup

**Fig. 1:**
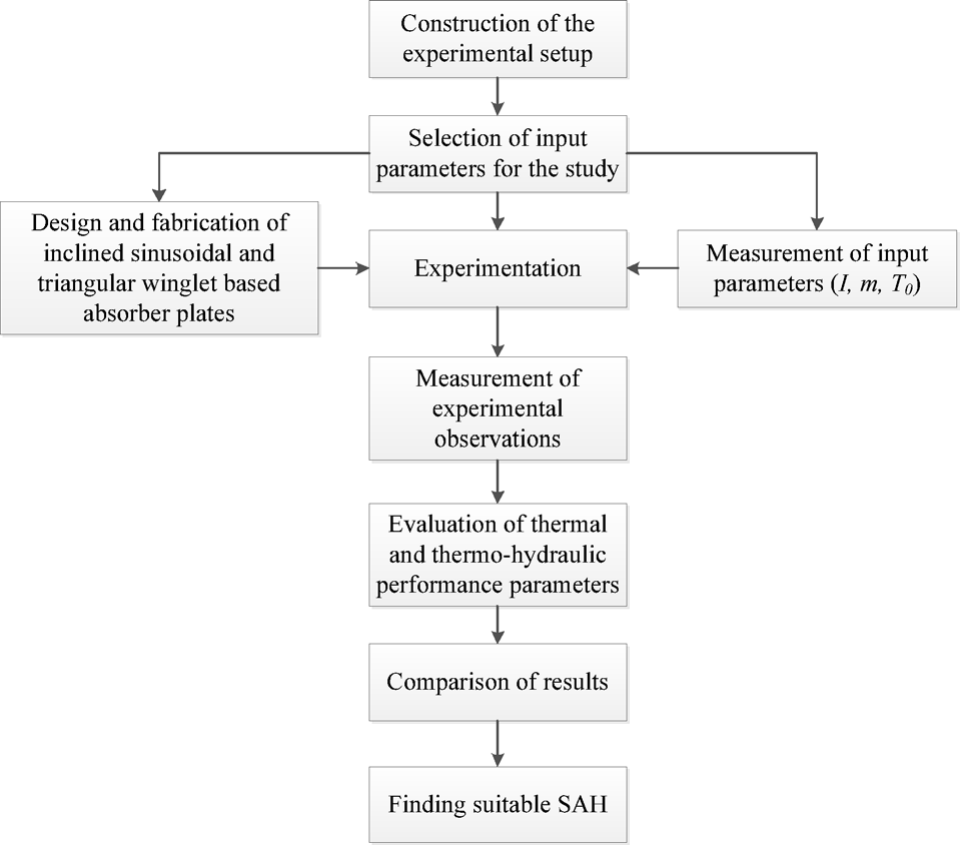
Methodology followed in the study.

Two SAH systems were built and installed in Kovilpatti, South Tamil Nadu (latitude: 9.1484° N, longitude: 77.8322° E) in compliance with ASHRAE standard 93, and their specifications are presented in Table 2. To optimize solar energy absorption, the SAH was purposefully installed in the North-South. The necessary provision has been provided in the experimental setup to attach the absorber plate when needed and can be replaced with another absorber plate. The absorber plates are fabricated with the inclined sinusoidal and triangular-shaped winglets separately and are attached to the experimental setup during the experimentation. When the absorber plate with the inclined sinusoidal winglet is fixed in the SAH it forms the ISWSAH and the plate with the inclined triangular winglet is fixed in the SAH it forms the IWSAH. A blower with a 2800 rpm and 1 HP (750 W) power rating was utilized to blow the air. The performance of the built collector plate in the SAH was investigated for the air flow rates of 0.01 kg/s, 0.02 kg/s, and 0.03 kg/s. The methodology for this system study is presented in Fig. 1. For calculating the air flow rate, the input tube’s cross-sectional area and air density were used. The airflow rate is controlled by a blower-integrated control valve.

Every configuration of the solar air heaters (IWSAH & ISWSAH) was tested separately on different days because of the experimental limitations in modifying the setup. Experiments were performed on each configuration over several testing days (9 days for IWSAH & 9 days for ISWSAH) to attain reliable results. Every flow rate tested 3 different days for both configurations. To make a fair comparison among the two designs, only days with a clear sky and equal solar radiation were considered for evaluation. The selection of days considered a minimum average global solar irradiance of approximately 300 W/m² over the test days with a maximum allowable variation of not more than ± 5% in hourly solar radiation.

**Table 2:** Solar air heater specification.

Parameters	Ranges
Inlet & outlet dia	40 mm
Length of inlet & outlet pipe	150 mm
Area of the absorber Transparent Thickness	1500 mm 5 mm
Aluminium plate	1.2 mm
Absorber dimensions Insulation Thickness	1.5 × 1 5 mm
SAH Dimensions Baffle Height Baffle Angle	1550*1050*100 mm 80 mm 60°

**Fig. 2:**
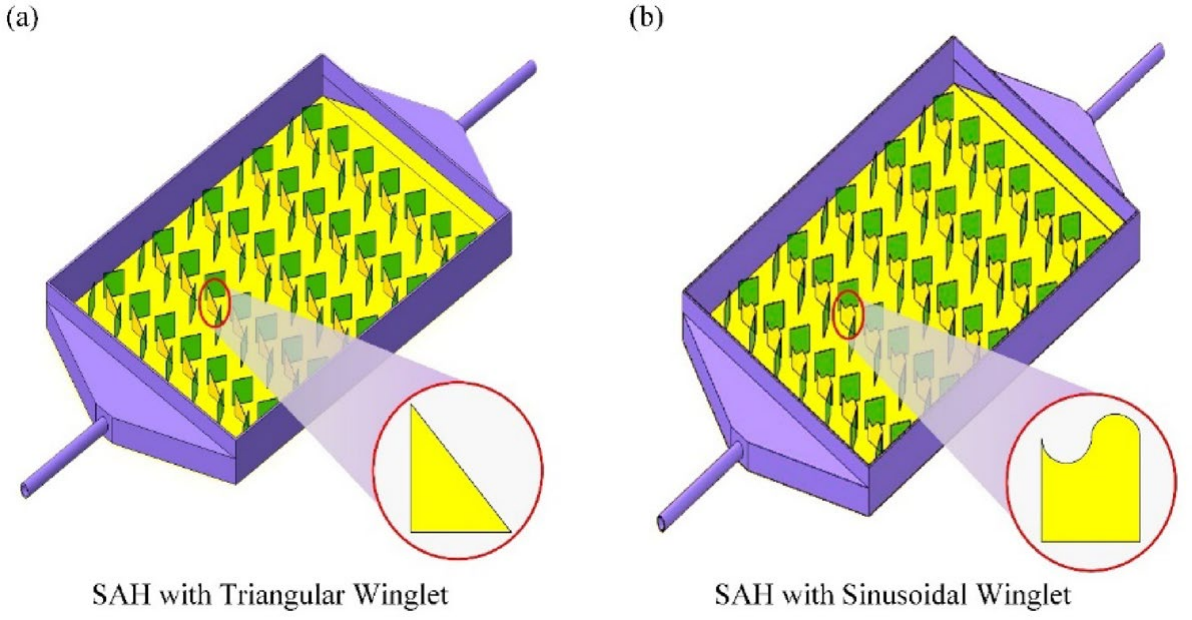
Schematic layout of (**a**) IWSAH and (**b**) ISWSAH.

**Fig. 3:**
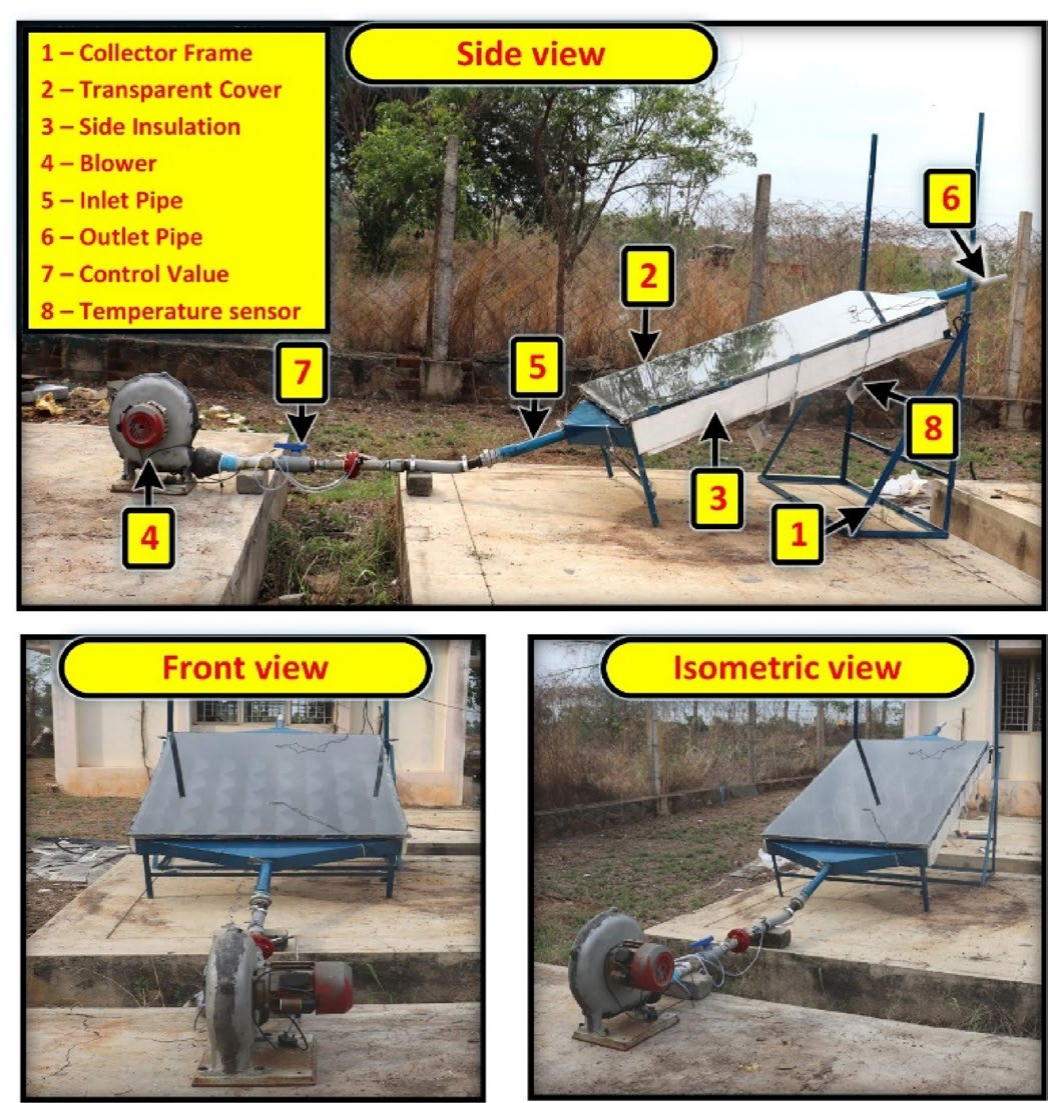
Photographic view of the installed air heater setup.

### Instrumentation

A thermocouple (Type-K) was used for temperature measurements at the required points of the SAH. The data gathering system recorded the temperature of the glass at two different positions, the temperature of the collector plate at four different points, and the temperature of the air outlet within the SAH every thirty minutes between 8 a.m. and 6 p.m. Figures 2a and b show the design configuration of the IWSAH and ISWSAH SAHs used in this investigation, and Fig. 3 shows a photographic image of the experimental apparatus. The details of the instruments utilized are described in Table 3.

**Table 3:** Instrumentation details used in experimentation.

Instrument	Range	Accuracy
Handheld solarimeter	0–2500 W/m^2^	±1 W/m^2^
Handheld Anemometer	0–35 m/s	± 0.05 m/s
Thermocouple wires	0 to 600 °C	± 1 °C
Digital temperature indicator	0–500 °C	± 1 °C

Uncertainty propagation of derived performance parameters was performed using the standard root-sum-square method. Uncertainty in pumping power was derived by propagating pressure drop, air density, volumetric flow rate, and blower efficiency uncertainties. In a similar way, the thermo-hydraulic efficiency uncertainty was calculated as the root-sum-square of thermal efficiency, friction factor, and pumping power uncertainties, considering their functional dependence. Using such an approach, the overall uncertainty in pumping power was estimated to be within ± 4.2%, while the uncertainty in thermo-hydraulic efficiency was found within ± 5.1%, which is within the uncertainties reported in similar experimental studies on solar air heaters.

## Energy analysis

### Energy performance

In this investigation, experimental results include air temperatures at inlet and outlet, absorber plate temperatures, solar irradiance, air mass flow rate, and pressure drop over the test rig. The above parameters measured will allow assessment of thermal and fluid dynamic performances of the solar air heater. The convective coefficient will be computed using the gain in heat transfer and the logarithmic mean temperature difference existing between the absorber plate and moving air. The pressure differential over the duct will be measured using a differential pressure gauge, where using standard fluid dynamics equations based on geometric parameters of the duct and Re numbers, friction factor evaluation will follow. The blower power consumption will be computed using parameters such as pressure differential and air volumetric flow rate considering blower efficiency.

The following formulae are used to assess the suggested SAH’s energy efficiency^25–27^.

1 $$\:{Q}_{c}={A}_{s}{F}_{R}\left[I\right(\tau\:\alpha\:{)}_{e}-{U}_{L}({T}_{i}-{T}_{a})]$$

Where, *Q*_*c*_ - heat gained from the plate (W), $\:{A}_{s}$ - surface area of absorber plate (m^2^),$\:\:{F}_{R}$ - absorber heat removal factor, $\:I$ - solar radiation (W/m^2^), $\:{\left(\tau\:\alpha\:\right)}_{e}$ - effective transmittance and absorbance product, $\:{U}_{L}$- overall heat transfer coefficient (W/m^2^/K), $\:{T}_{i}$ - air inlet temperature (K) and $\:{T}_{a}$ = ambient temperature (K).

The absorber’s heat removal may be calculated using,

2 $$\:{F}_{R}=\frac{\dot{m}{C}_{p}}{{U}_{L}{A}_{s}}\left[1-{exp}\left\{-\frac{{F}^{{\prime\:}}}{{U}_{L}{A}_{s}}\right\}\right]$$

where, ṁ - rate of airflow (kg/s), Cp - air’s specific heat (kJ/kgK), ρ - air’s density (Kg/m^3^), F´- factor of absorber efficiency and it can be estimated as follows,

3 $$\:{F}^{{\prime\:}}=\frac{{h}_{fc}{h}_{r}+{h}_{fp}{U}_{t}+{h}_{fp}{h}_{r}+{h}_{fc}{h}_{fp}}{({U}_{hc}+{h}_{r}+{h}_{fc})({U}_{b}+{h}_{r}+{h}_{fb})-{h}_{r}^{2}}$$

where, U_L_ - overall heat transfer coefficient and it can be calculated from below Eq. (4)^42^

4 $$\:{U}_{L}=\frac{({U}_{t}+{U}_{b})({h}_{fc}{h}_{fp}+{h}_{fc}{h}_{r}+{h}_{fp}{h}_{r})+{U}_{t}{U}_{b}({h}_{fc}+{h}_{fp})}{{h}_{fc}{h}_{r}+{h}_{fp}{U}_{t}+{h}_{fp}{h}_{r}+{h}_{fc}{h}_{fp}}$$

where, U_t_, U_b_ & U_s_ defines top, bottom and side loss coefficient.

5 $$\:U_{t} = \frac{{T_{g} - T_{a} }}{{\frac{1}{{A_{s} \left( {h_{r} + h_{w} } \right)}}}}$$

where, $\:{T}_{g\:}$- glass cover temperature (K),$\:{\:h}_{r}$ - radiation heat transfer coefficient in top (W/m^2^ K).

The radiation heat transfer can be estimated using,

6 $$\:h_{r} = \frac{{\sigma \:(T_{{pm}}^{4} - T_{c}^{4} )}}{{\left( {\frac{1}{{ \in _{p} }} + \frac{1}{{ \in _{c} }} - 1} \right)(T_{{pm}} - T_{c} )}}$$

σ is the Stefan Boltzmann constant (5.667 × 10^− 8^ W/m^2^ K^4^).

Wind heat transfer coefficient at the top cover (h_w_) is as follows,

7 $${h_w}=5.67+3.86 \times {V_w}$$

where, V_w_ - velocity of wind (m/s).

The total useful power of energy gained by the system can be evaluated through,

8 $$\:{Q}_{u}=\dot{m}{C}_{p}({T}_{o}-{T}_{i})$$

9 $$\:\dot{m}=\rho\:AV$$

Furthermore, the following formula can be used to assess a SAH’s efficiency.

10 $$\:{\eta\:}_{e}=\frac{{Q}_{u}}{I{A}_{c}}$$

The SAH pumping power estimated using,

11 $$\:{p}_{f}=\frac{\dot{m}\varDelta\:p}{{\rho\:}_{a}}$$

where, Δp is the pressure drop which can be evaluated using

12 $$\:\varDelta\:P=\frac{2f\rho\:l{v}^{2}}{{D}_{e}}$$

where, D_e_ is the hydraulic diameter, and it is evaluated using

13 $$\:{D}_{e}=\frac{4{A}_{c}}{P}$$

The SAH’s heat transfer coefficient needs to be calculated using Re value, which has been evaluated using

14 $$\:\mathrm{Re}=\frac{\rho\:V{D}_{e}}{\mu\:}$$

Since SAHs use blowers to function, it is imperative to assess the thermo-hydraulic efficiency by taking pumping power into account. This can be done using

15 $$\:{\eta\:}_{H}=\frac{{Q}_{u}-{p}_{f}}{I{A}_{c}}$$

### Economic and environmental analysis

This analysis can be used to determine the return on investment for an SAH system. It also makes it easier to choose the best SAH based on the results of environmental and economic performance analysis. The economic and environmental behavior of SAHs is evaluated by the embodied energy of the SAH system. Based on a 20-year lifespan assumption, the system manufacturing capital investment under 10% interest is ₹26,000 for IWSAH and 25,600 for ISWSAH. The annual maintenance cost will be approximately 10% of the fixed cost. Additionally, it is estimated that the salvage value will equal 20% of the original capital cost. The formulas listed below are used to evaluate SAH’s environmental performance and the assumption values are obtained from references^27–29^.

16 $$\:\mathrm{Energy\:payback\:time}\left(EPBT\right)=\frac{{E}_{in}}{{E}_{out}}$$

where, E_in_ - Embodied energy obtained from SAH in kWh, and E_out_ is the energy output in kWh/yr.

17 $$\:\mathrm{Energy\:production\:factor}\left(EPF\right)=\frac{{E}_{out}}{{E}_{in}}\times\:{T}_{L}$$

where, T_L_ is the lifespan of SAH in years

18 $$\:\mathrm{Life\:Cycle\:Conversion\:Efficiency\:}\left(\mathrm{LCCE}\right)=\frac{({E}_{\mathrm{out}}\times\:{T}_{L})-{E}_{\mathrm{in}}}{{E}_{\mathrm{sol}}\times\:{T}_{L}}$$

19 $$\:\mathrm{C}{\mathrm{O}}_{2}\mathrm{\:Emission}={E}_{\mathrm{in}}\times\:1.58$$

20 $$\:\mathrm{NO\:Emission}={E}_{\mathrm{in}}\times\:0.005$$

21 $$\:\mathrm{S}{\mathrm{O}}_{2}\mathrm{\:Emission}={E}_{\mathrm{in}}\times\:0.012$$

22 $$\:{CO}_{2\:}mitigation\:\left(kg\:of\:{CO}_{2}\right)={E}_{out}*1.58$$

The economic performance of the SAH is calculated using the equations below.

23 $$\:\mathrm{Cost\:of\:yield},C=\frac{TAC}{TAY}$$

where TAY stands for Total Accumulated Yield and TAC stands for Total Annual Cost in Indian Rupees.

24 $$\:TAC=FAC+YMC-YSC$$

where YMC is for Yearly Maintenance Cost, YSC for Yearly Salvage Cost of SAH, and FAC stands for Fixed Annual Cost in Indian Rupees.

25 $$\:FAC=PCC\times\:CFR$$

where, PCC is the Primary Capital Cost in INR, and CRF is the Capital Recovery Factor

26 $$\:CFR=\frac{i{\left(1+i\right)}^{n}}{{\left(1+i\right)}^{n}+1}$$

where i is the interest rate in %, and n is the lifespan of SAH in years

27 $$\:YMC=0.05\times\:FAC$$

28 $$\:YSC=S\times\:SFF$$

where ASC is the annual salvage cost, S is the salvage cost in Indian rupees, and SFF is the sinking fund factor.

29 $$\:S=0.2\times\:PCC$$

30 $$\:SFF=\frac{i}{{\left(1+i\right)}^{n}+1}$$

### Error analysis

The following equations were used in error or uncertainty analysis to evaluate when mistakes were made during the testing procedure and whether the equipment used had errors. The obtained error value is shown in Table 4^30,31^.

Uncertainty in mass flow rate is evaluated using,

31 $$\:\frac{{\omega\:}_{\dot{m}}}{\dot{m}}={\left[{\left(\frac{{\omega\:}_{{c}_{d}}}{{c}_{d}}\right)}^{2}+{\left(\frac{{\omega\:}_{A}}{A}\right)}^{2}+\frac{1}{4}{\left(\frac{{\omega\:}_{{p}_{1}}}{{p}_{1}}\right)}^{2}+{\left(\frac{{\omega\:}_{\varDelta\:P}}{\varDelta\:P}\right)}^{2}+{\left(\frac{{\omega\:}_{{T}_{1}}}{{T}_{1}}\right)}^{2}\right]}^{1/2}$$

Assuming that uncertainties in temperature and pressure difference measurements are the only sources of uncertainty, the previous equation reduces as follows.

32 $$\:\frac{{\omega\:}_{\dot{m}}}{\dot{m}}={\left[{\left(\frac{{\omega\:}_{\varDelta\:P}}{\varDelta\:P}\right)}^{2}+{\left(\frac{{\omega\:}_{{T}_{1}}}{{T}_{1}}\right)}^{2}\right]}^{1/2}$$

When calculating the thermal performance, uncertainty arises and it is calculated using

33 $$\:\frac{{\omega\:}_{\eta\:}}{\eta\:}={\left[{\left(\frac{{\omega\:}_{m}}{m}\right)}^{2}+{\left(\frac{{\omega\:}_{T}}{T}\right)}^{2}+{\left(\frac{{\omega\:}_{I}}{I}\right)}^{2}\right]}^{1/2}$$

**Table 4:** Uncertainty of the observed and calculated values.

Parameter	Error
Efficiency (%)	± 6%
Solar Radiation (W/m^2^)	± 0.5
Flow rate (kg/s)	± 4%
Temperature (°C)	± 2.8
Wind velocity (m/s)	± 0.2

## Results and discussion

**Fig. 4:**
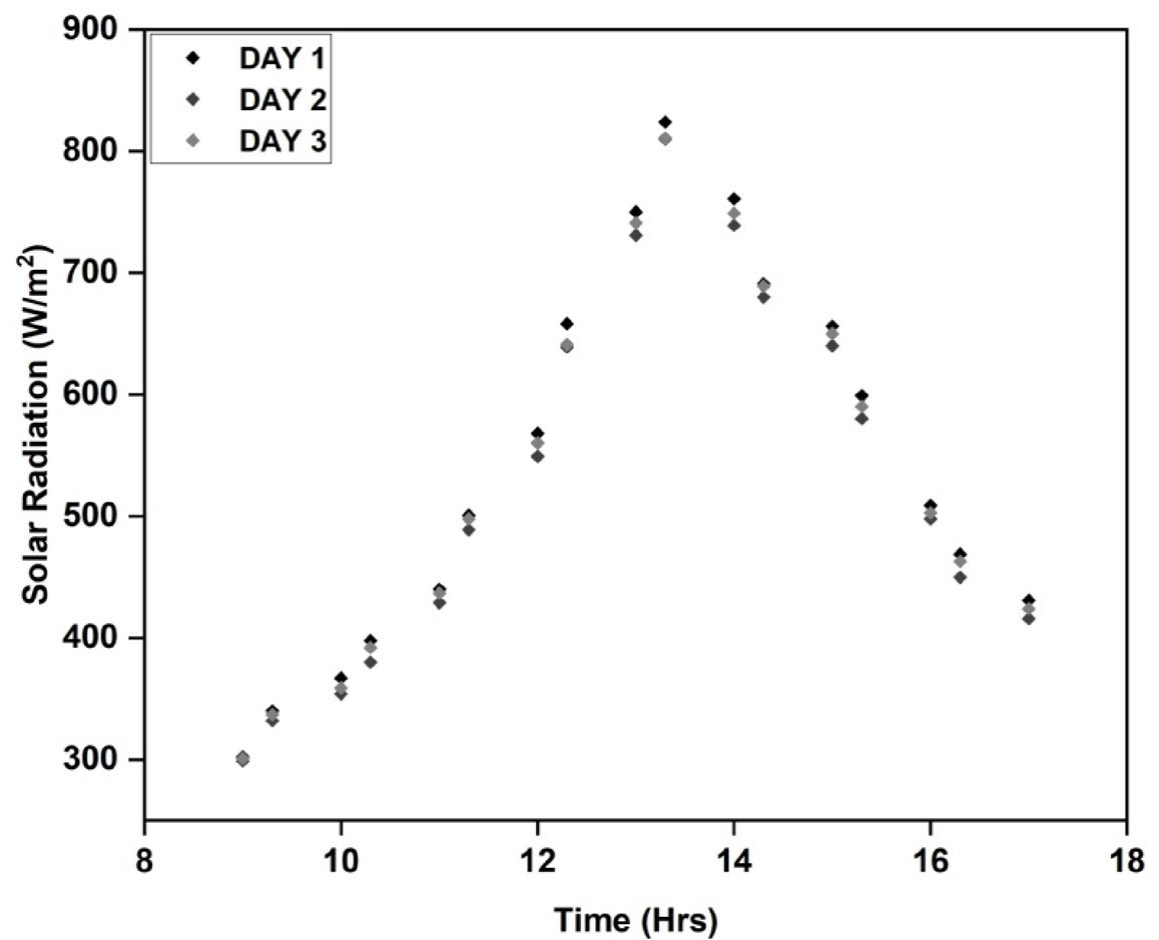
Radiation during experiment days.

**Fig. 5:**
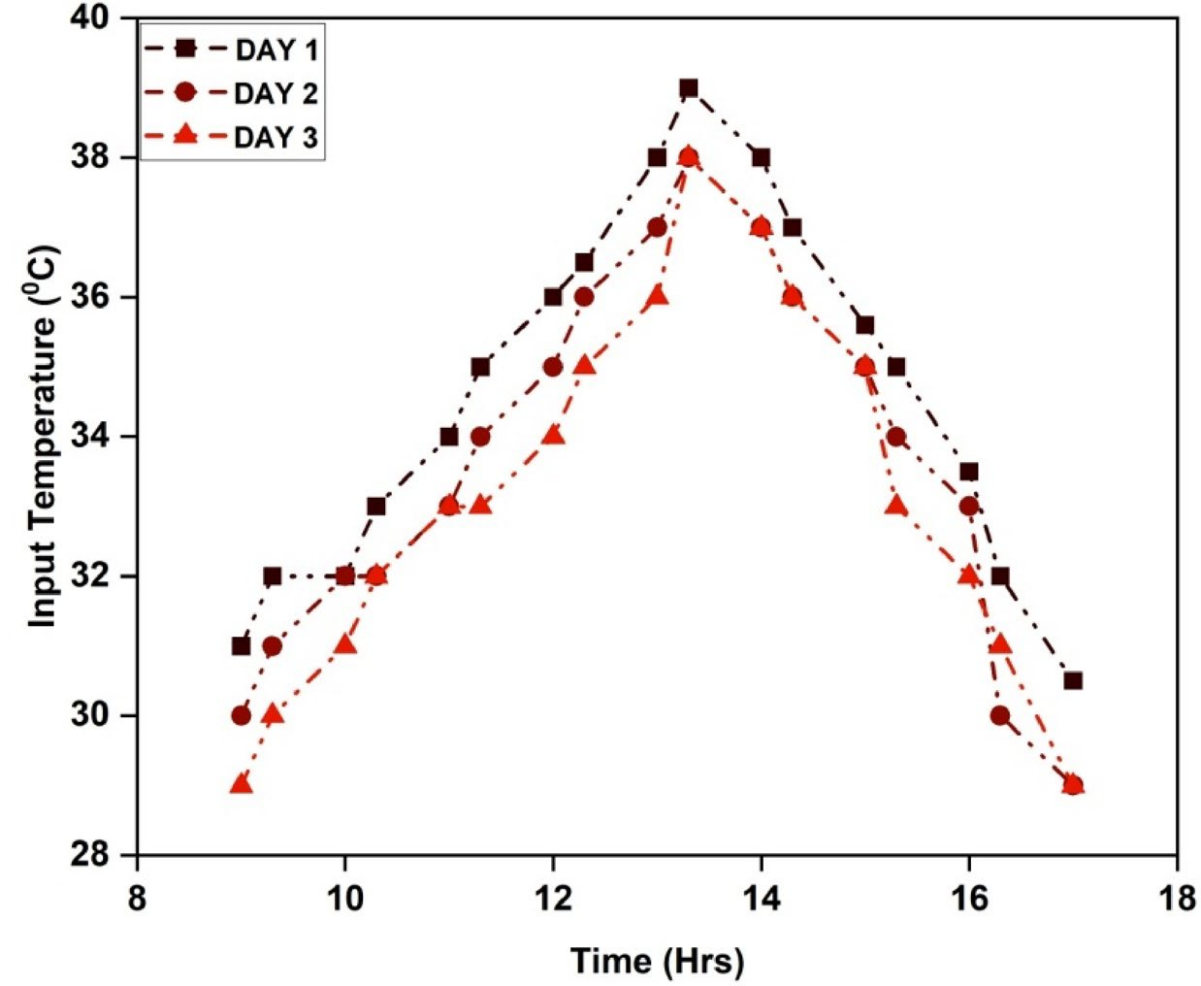
Inlet air temperature.

Since solar radiation is SAHs’ primary energy source, exact measurement is essential to the growth, advancement, and improvement of these systems. Accurate evaluation of solar radiation data is necessary for determining the viability of renewable energy sources and predicting the effectiveness and functionality of solar-assisted heating systems (SAHs). For most SAH studies to be successful and produce observable effects, it is generally believed that a minimum solar radiation level of between 300 and 500 W/m^2^ is ideal. On clear, sunny days, there is usually a moderate-to-high amount of solar radiation reflected in this value. Figure 4 depicts the solar radiation recorded on the three days of the experiment at the site, which peaks in the morning and afternoon and then gradually decreases until the evening. During this period, the radiation levels vary between 299 and 824 W/m^2^, which is sufficient to assess the SAH’s efficiency^32^. It is reasonable to compare the performance because the variations in solar radiation levels throughout the course of the testing days are just 5%. When the temperature of the input air is measured, it becomes easier to study the SAH’s energy transfer process. The temperature variance between the collector plate and the outside air influences the heat transfer rate. To increase the effectiveness of the system and design, it is critical to understand temperature differences. Figure 5 depicts the measured air inlet temperature of the experiment. The temperature of the intake increases during the morning and afternoon and thereafter decreases till twilight. The temperature range of the experiment spans from 29 to 39 °C, whereas the average temperature at the entrance falls between 33 and 35 °C.

**Fig. 6:**
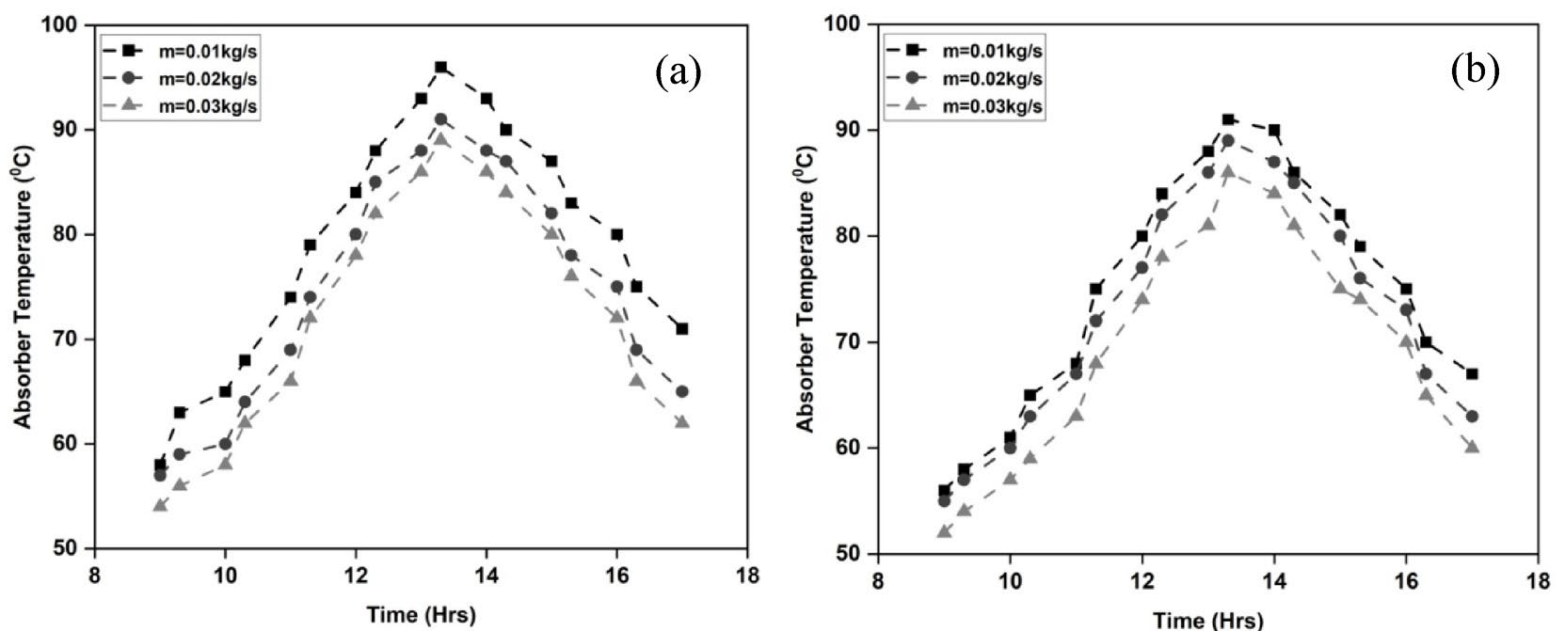
Collector plate temperature in (**a**) ISWSAH and (**b**) IWSAH.

The absorber plate and glass plate temperatures have an impact on the heat transfer in SAH. These temperatures can be used to analyze how well the system converts solar radiation into thermal energy. Better solar energy absorption is correlated with higher temperatures on the absorber plate. Typically, some of the thermal energy produced by the absorber plate’s contact with the sun is transmitted to the glass cover. The collector plate generates higher temperatures due to the concentration of heat, and the airflow inside the SAH limits the complete passage of heat from the absorber to the glass^33^. Figure 6a and b display the absorber plate temperatures of the two air heaters under investigation. The relationship between higher air flow rate and lower plate temperature is depicted in the plots. A higher amount of contact between the air and the collector plate is caused by the increased air flow rate. As a result, convective heat transfer increased between the air and collector plate and the temperature of the plate fell due to enhanced air circulation. For the airflow under study, the average collector plate temperatures are 75.2, 72.6, and 68.7 °C for the IWSAH and 79, 74.1, and 70.9 °C for the ISWSAH. It demonstrates that the IWSAH has significantly more heat transmission from the collector plate than the ISWSAH.

**Fig. 7:**
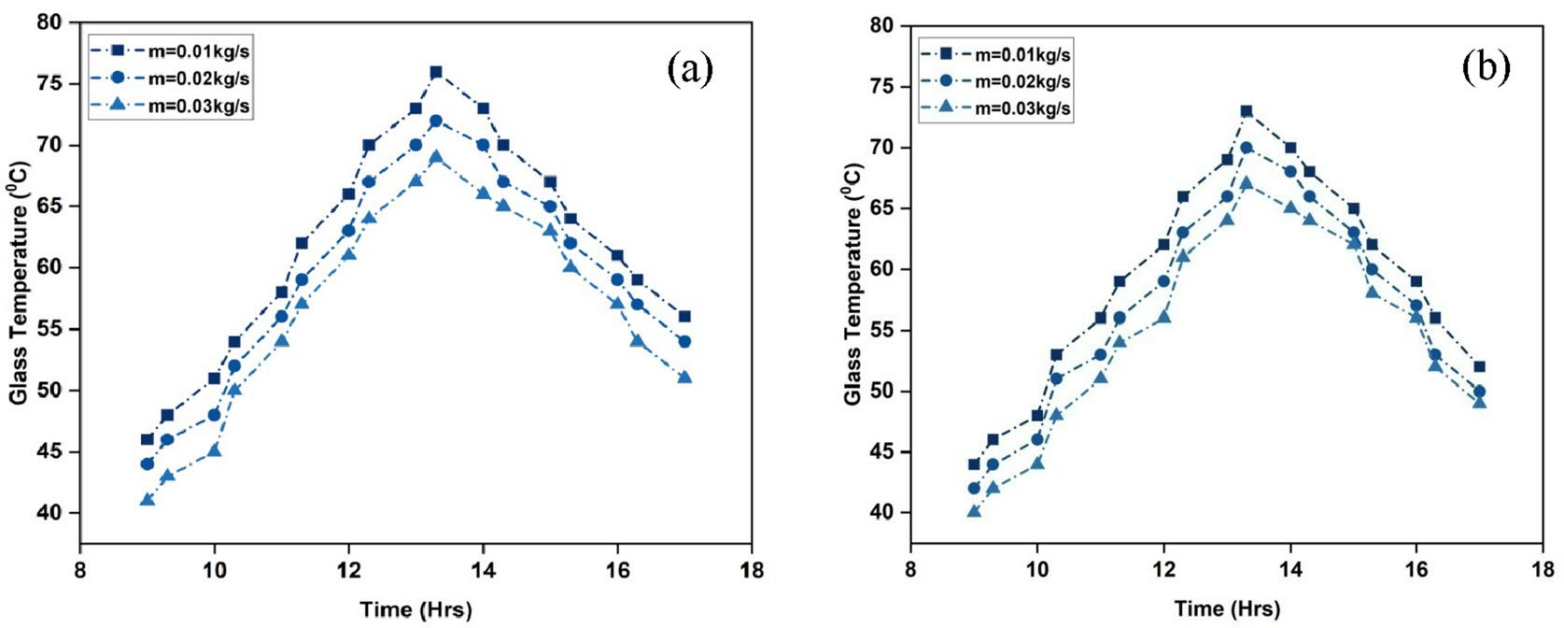
Variation of glass temperature absorbed in (**a**) ISWSAH and (**b**) IWSAH.

Figure 7a and b show the glass plate’s temperature over the course of the investigation days. Although allowing sunlight can pass through, the transparent glass cover is designed to minimize heat loss caused by radiation. Glass is less photosensitive to solar light than the absorber plate. It keeps a lower temperature than the absorber plate because of absorbs less heat from the incoming radiation. The glass plate covering it may lose heat in reverse if the absorber plate and cover are at different temperatures. By reducing the temperature of the glass plate and reducing heat loss, increasing the air mass flow rate reduces the temperature differential between the absorber plate and the glass plate^34^. In the case of ISWSAH, the average temperature of the glass plate is 62 °C, 58.6 °C, and 55.9 °C, thus, when the flow rates are 0.01, 0.02, and 0.03 kg/s. When comparing the tested flow rates, IWSAH shows slightly lower average temperatures of the glass plate, specifically 59.1 °C, 56.4 °C, and 54.2 °C. This demonstrates that there is a higher level of heat transfer from the Internal Wall Surface Average Heat (IWSAH) to the surrounding air.

**Fig. 8:**
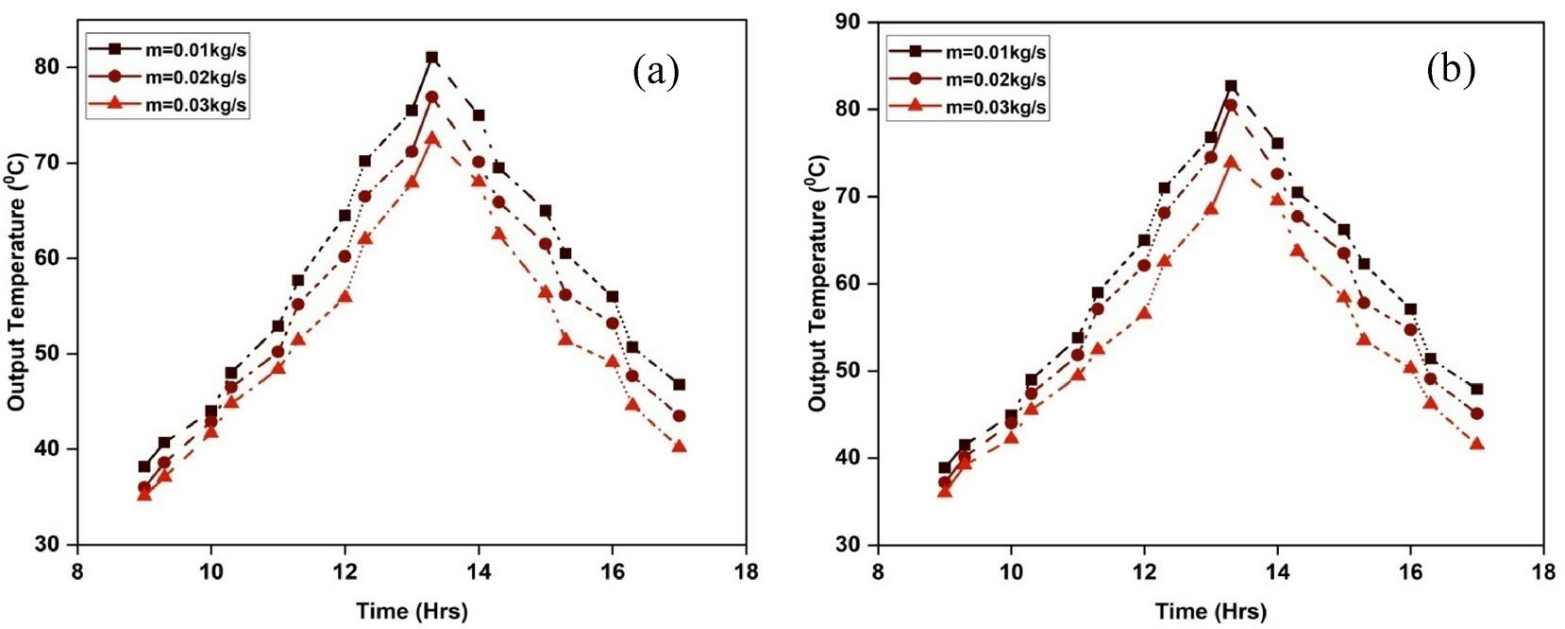
Variation of output air temperature in (**a**) ISWSAH and (**b**) IWSAH.

Various SAH designs are evaluated based on their output temperature. It serves to determine how effectively different systems perform under comparable operating circumstances. The observed output air temperatures for the two air heater designs are shown in Figs. 8a and b. According to the data analysis, IWSAH’s output temperature is noticeably greater than ISWSAH’s. The statistics also show that a rise in air mass flow rate causes the air temperature to decrease^35,36^. Higher air flow rates cause the air to accelerate its time to attain thermal equilibrium with the absorber plate. It may be inferred from this that the heat released by the absorber plate cools the solar air heater (SAH) since the air was unable to absorb it completely. Furthermore, when the airflow rate rises, more air passes over the absorber plate, improving the system’s convective heat transfer. The output temperature drops as a result of the absorber plate absorbing more heat due to increased convective cooling.

**Fig. 9:**
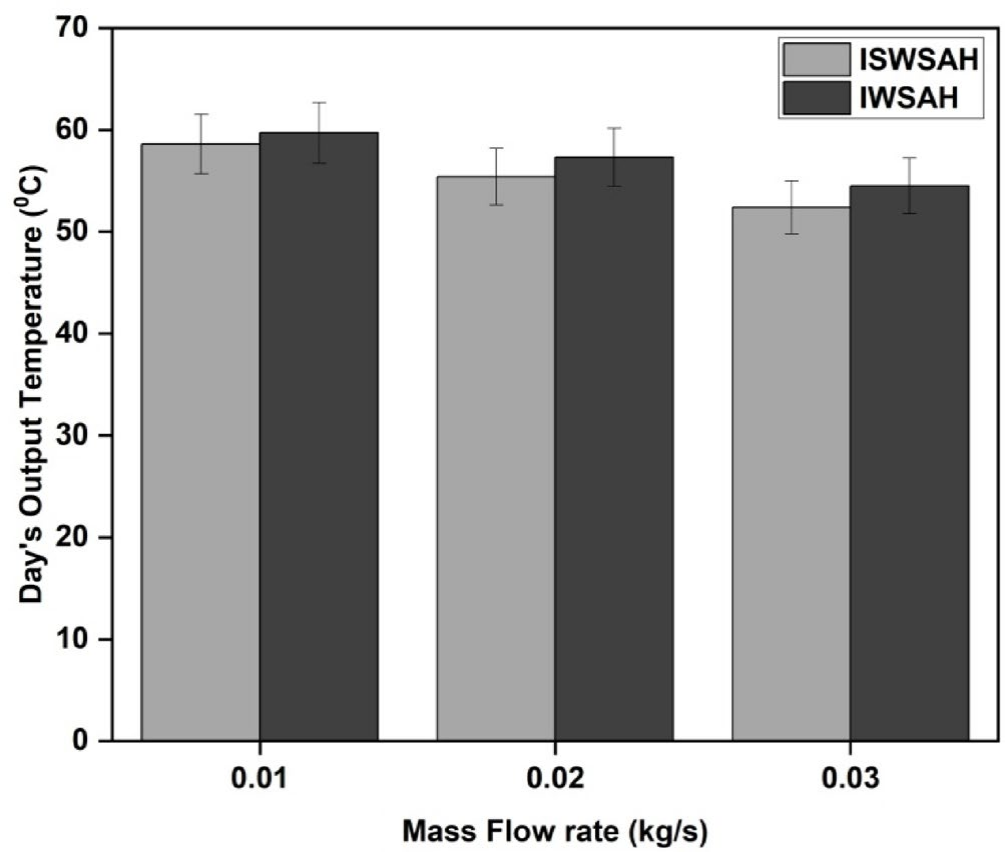
Day’s average output temperature in both SAHs.

The Figure shows that SAH outputs have their highest temperatures in the afternoon. IWSAH can attain a maximum temperature of 82.7 °C, but ISWSAH can only reach a minimum temperature of 81.1 °C when it operates at a flow rate of 0.01 kg/s. Figure 9 illustrates the average output temperatures of both solar air heating (SAH) systems. The average output temperatures for ISWSAH are 58.6 °C, 55.4 °C, and 52.4 °C for flow rates of 0.01 kg/s, 0.02 kg/s, and 0.03 kg/s, respectively. According to IWSAH, the mean output temperatures at identical flow rates were 60.7, 57, and 53.5 °C. An intriguing observation is that IWSAH consistently generates temperatures that are 3.5%, 2.8%, and 2.09% higher than ISWSAH. The results indicate that IWSAH exhibits superior internal turbulence and heat transport in comparison to ISWSAH.

**Fig. 10:**
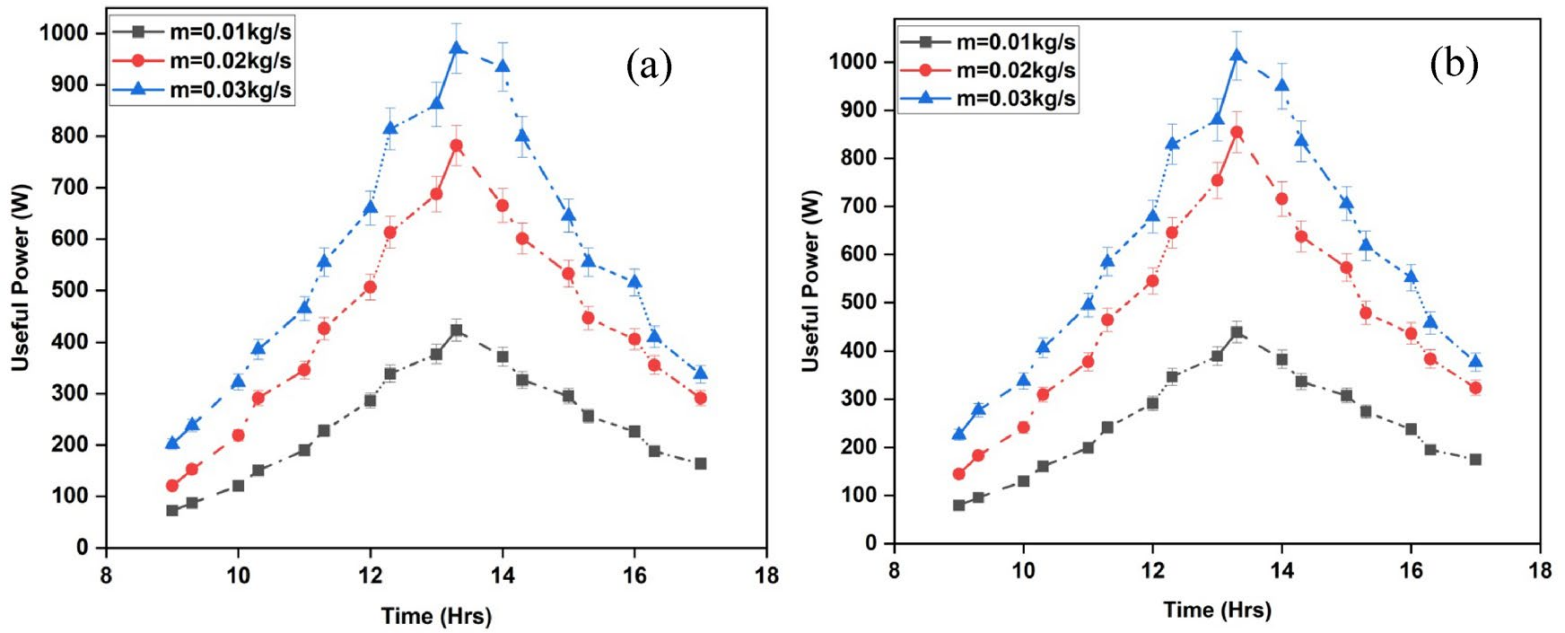
Variation of energy gained by (**a**) ISWSAH and (**b**) IWSAH.

The energy gain or useful power is a common parameter for comparing various designs or technologies of solar air heaters (SAH). Objective evaluations can be conducted to determine the efficiency and appropriateness of a system for specific users or settings. Additionally, it aids in assessing the overall effectiveness and efficiency of the SAH. Figures 10a and b illustrate the energy gain derived from the system analysis. The energy gain has a clear pattern of increasing until midday and then decreasing, which is influenced by the radiation route and system temperature. The system’s internal temperature differential significantly affects the behavior of this energy gain. While raising the air mass flow rate, both SAH systems offer a considerable energy gain. When the airflow rate is larger, the SAH can process more air in each amount of time^37^. A greater amount of heat is transferred from the collector to the environment, increasing the amount of energy obtained. In addition, lower air retention times in the SAH are caused by higher airflow rates. Because of the collector’s shorter air exposure time, there is a lower chance of heat loss to the surroundings. The shorter time the air spends in the system causes the temperature differential between the absorber plate and the outflow air to grow, increasing the energy gain.

**Fig. 11:**
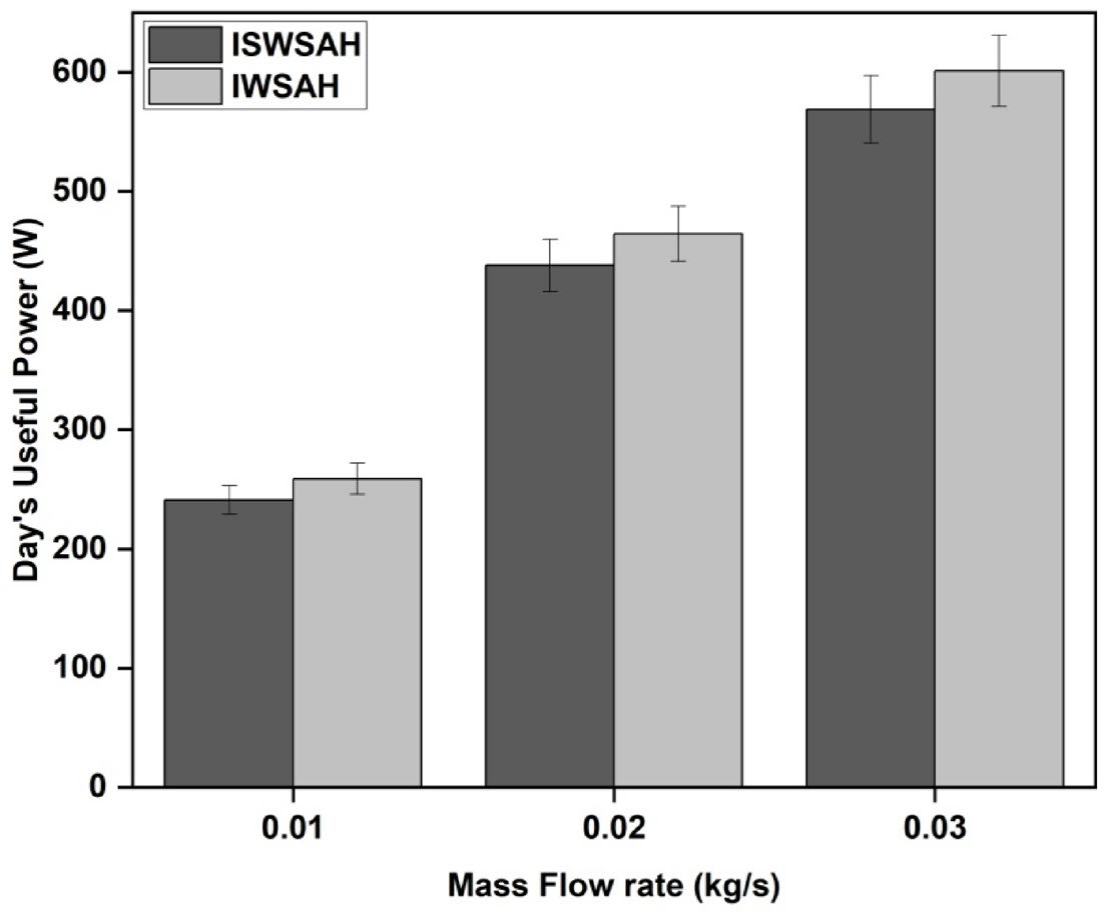
Average energy gained in both SAHs.

- At an afternoon flow rate of 0.03 kg/s, both SAH setups show significant energy gains, with 974.2 W for ISWSAH and 1012 W for IWSAH.
- The average energy gain presented in Fig. 11 shows that, for ISWSAH is 241.3 W, 437.83 W, and 575.65 W for flow rates of 0.01, 0.02, and 0.03 kg/s; for IWSAH, it varies between 251.84 W, 464.48 W, and 611.1 W.
- This indicates that IWSAH produces energy gains that are 4.36%, 6.08%, and 6.15% greater than those of ISWSAH for the equivalent flow rates under consideration.

**Fig. 12:**
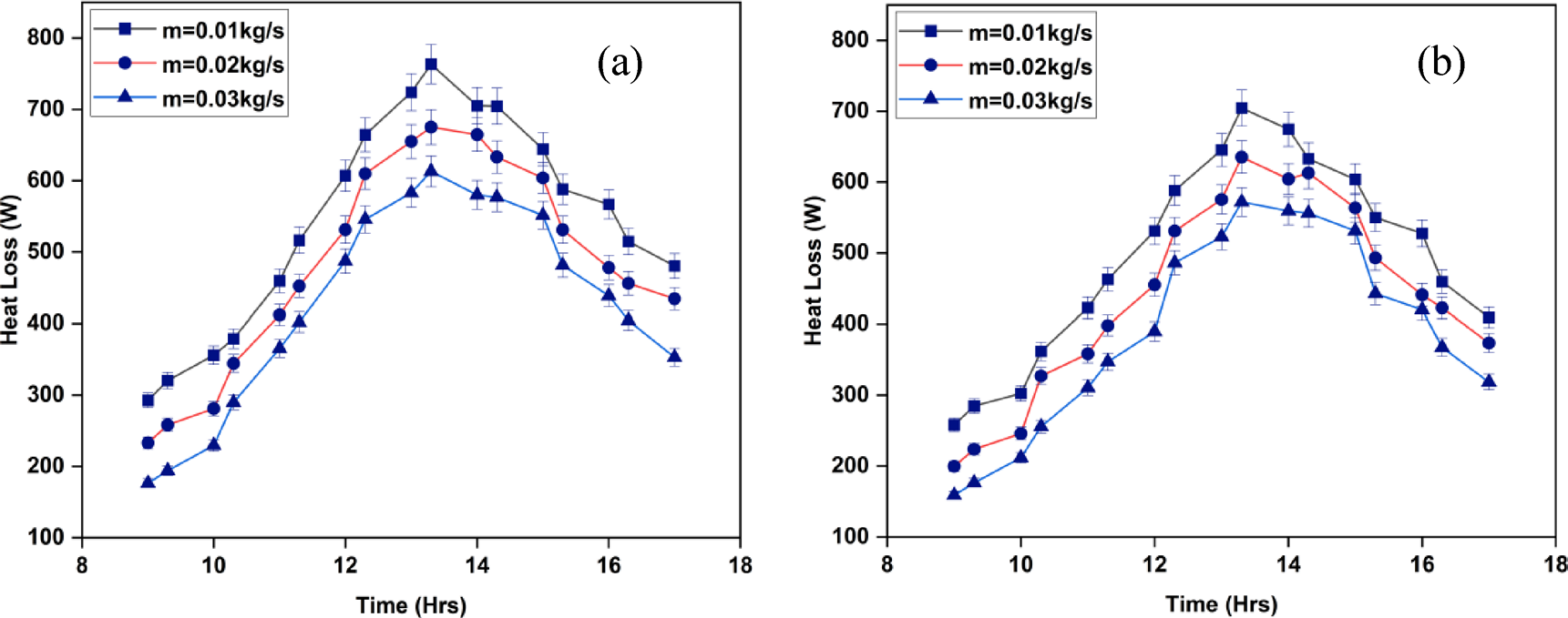
Variation of heat losses over the top surface for (**a**) ISWSAH and (**b**) IWSAH.

The heat dissipation from the top surface of the SAH results in energy loss. Our knowledge of how much solar energy is received, lost to the environment, and not used to heat the air is helped by the measurement of this heat loss. Figure 12a and b illustrate the heat loss that occurred throughout the SAH running time. As mass flow rates decrease, heat loss through the upper surface rises. Air volume passing across the absorber surface is regulated by its mass flow rate. There is less air in contact with the absorber when the mass flow rate is decreased. Reduced convective heat transmission causes an increase in heat retention on the upper surface of the heater, which raises heat losses. Moreover, upon reducing the mass flow rate, there can be regions on the absorber surface where the airflow is partially uncovered. Due to increased radiative heat loss and inadequate convective heat transmission, these exposed locations may see higher heat losses^38^.

**Fig. 13:**
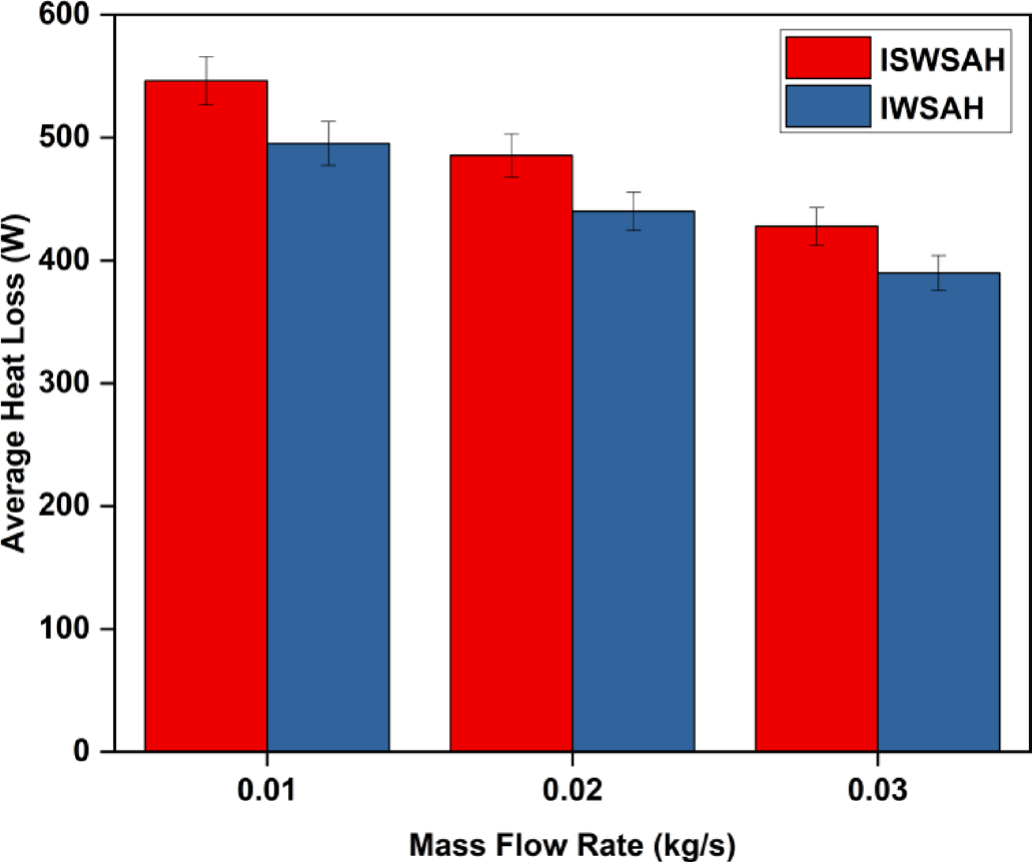
Average Heat Losses over the Top Surface for both setup.

The afternoon sees a significant increase in heat loss over the top surface, with maximum values for the IWSAH configuration being 704.2 W and the ISWSAH setup being 763.2 W. With flow rates of 0.01 kg/s, 0.02 kg/s, and 0.03 kg/s, respectively, the average heat losses across the top surface for the ISWSAH setup on test day are 534.2 W, 479.47 W, and 427.7 W, as illustrated in Fig. 13. The IWSAH system still loses an average of 389.7 W, 440 W, and 495.2 W of heat at the same flow rates. For IWSAH compared to ISWSAH, this is 7.87, 8.97, and 9.75% less. The IWSAH setup outperforms the ISWSAH setup in terms of performance and system heat loss, as demonstrated by these results.

**Fig. 14:**
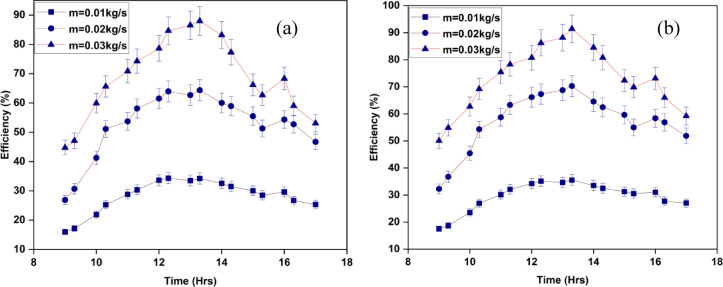
Variation of efficiency of (**a**) ISWSAH and (**b**) IWSAH.

The assessment of the reliability and achievement of SAHs heavily relies on efficiency. It offers valuable information for several objectives, including economic analysis, optimization, and decision-making. The efficacy of the proposed approach is shown in Figs. 14a and b. The efficiency of the system is closely correlated with the mass flow rates. An increased rate of air mass flow leads to a greater amount of air passing through the SAH, hence improving efficiency. As a result, the surface of the absorber experiences a high level of convective heat transfer with the surrounding air. Consequently, the system’s total efficiency increases when the absorber transfers a larger quantity of heat to the air^39]– [40^. To lessen the heat absorption phenomena in the interior air of the SAH, larger mass flow rates can be used to mitigate excessive heat. Heat soak occurs when the interior air of the heater becomes too hot and continues to emit heat for an extended period after sunset. Reduction of heat soak improves system efficiency overall.

**Fig. 15:**
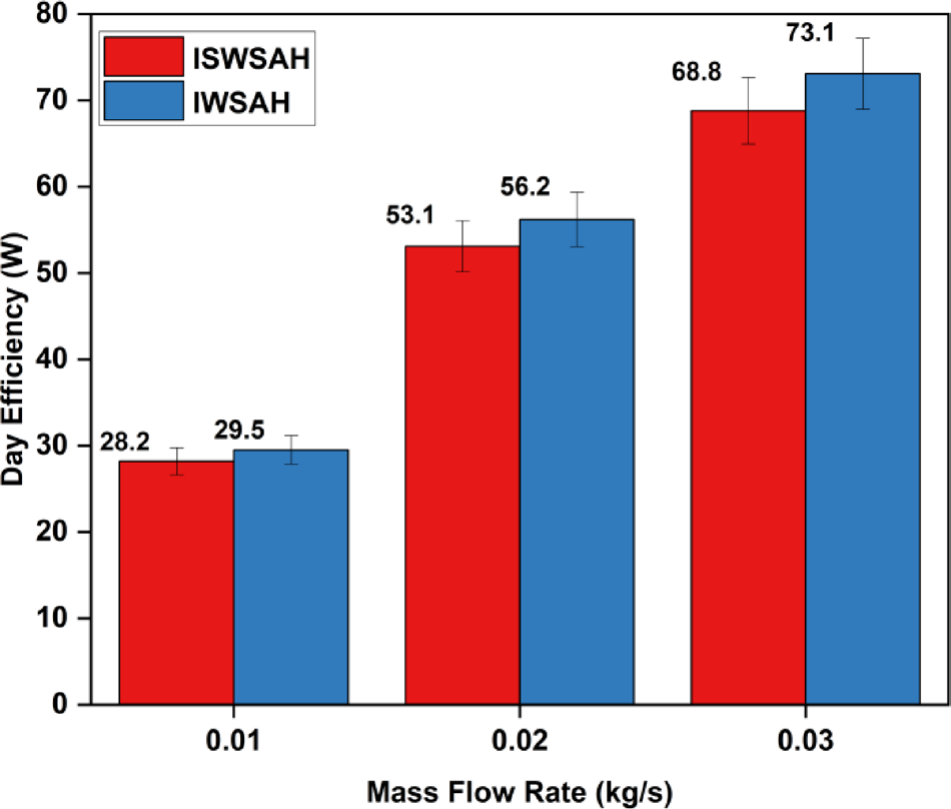
Day’s average efficiency for both SAHs.

- The ISWSAH reaches its maximum efficiency of 88% and the IWSAH reaches its maximum efficiency of 91% at a greater mass flow rate of 0.03 kg/s.
- The average IWSAH efficiencies for the experiment were 29.5%, 56.2%, and 73.1%, respectively, at flow rates of 0.01 kg/s, 0.02 kg/s, and 0.03 kg/s, as shown in Fig. 15.
- The ISWSAH has average efficiencies of 28.2%, 53.1%, and 68.8% for the same flow rates. The efficiency ranges of the IWSAH and ISWSAH are 4.6%, 5.8%, and 6.25% higher, respectively.

**Fig. 16:**
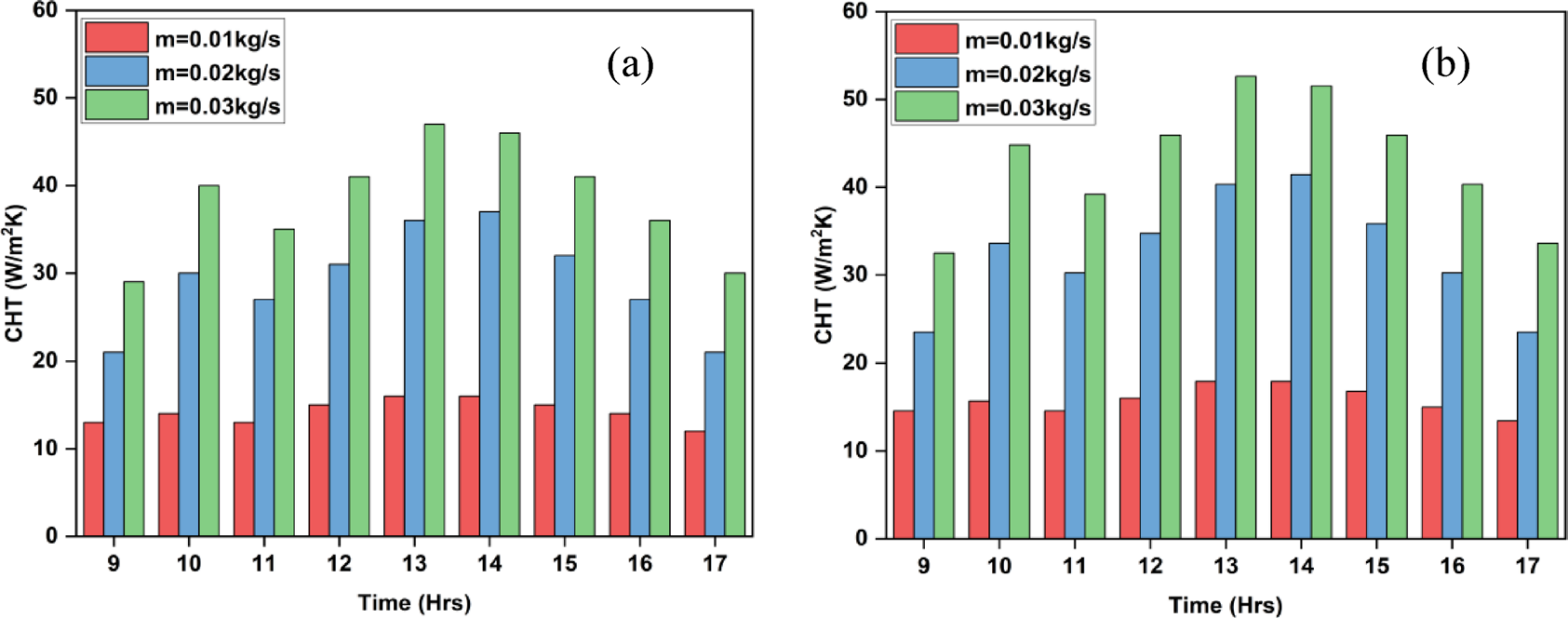
Variation of Heat Transfer Coefficient of (**a**) ISWSAH and (**b**) IWSAH.

**Fig. 17:**
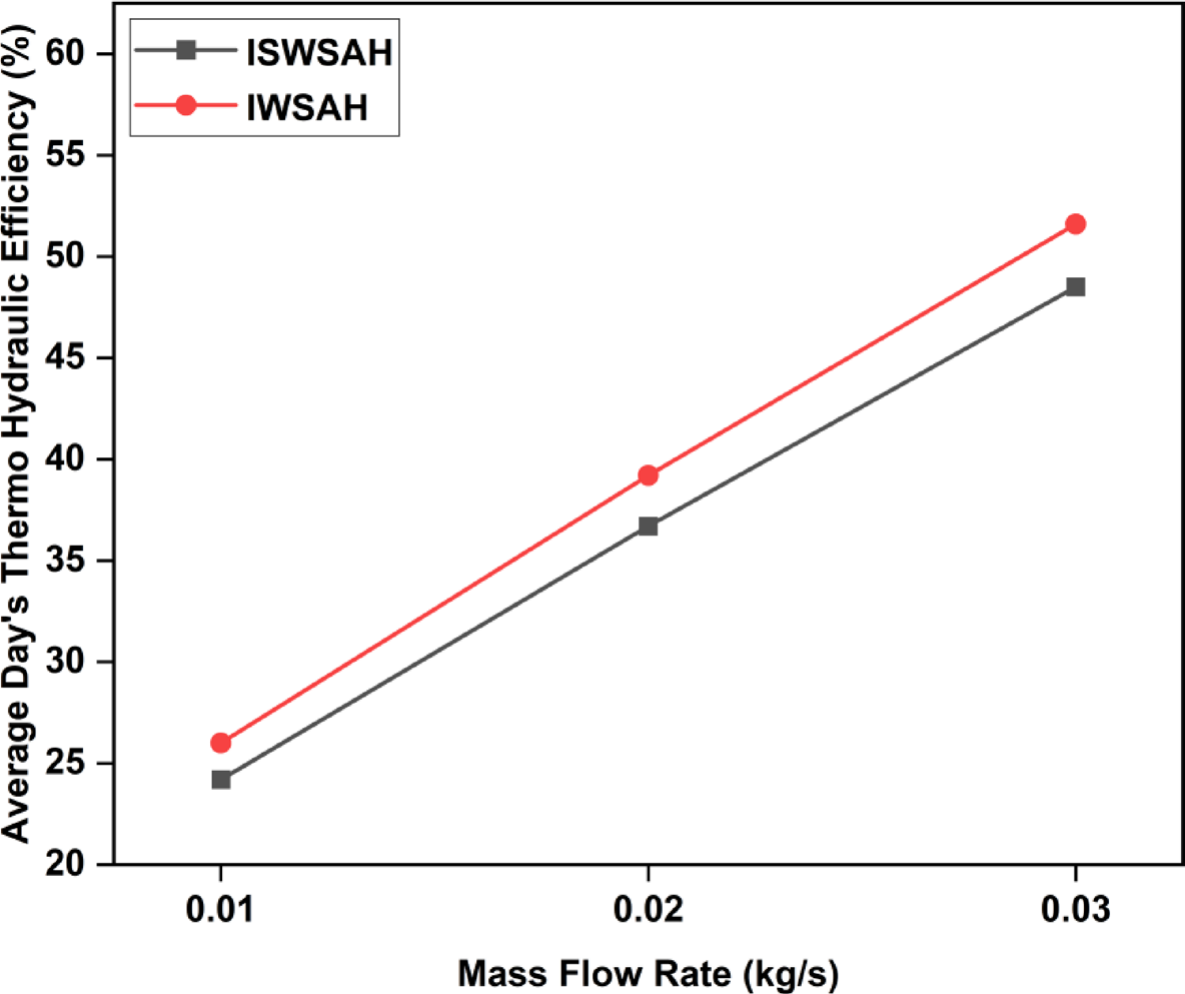
Average thermo-hydraulic efficiency for both SAH’s.

Analyzing the increase in heat transfer is essential for assessing the operation of a solar air heater, as it offers vital information about the efficiency of transporting heat from the solar collector to the air stream^41,42^. Heat transfer enhancement is increasing the rate at which heat is transmitted by making changes to the design of SAHs or employing certain procedures. Figure 16a and b illustrate how changing the airflow rate affects the convective heat transfer coefficient between the air in the inclined winglet (IWSAH) and inclined sinusoidal (ISWSAH) setups. From Fig. 17, the findings show that increasing the air mass flow rate for both SAHs causes a discernible increase in convective heat transfer (CHT). This observation is supported by the fact that increased airflow significantly quickens the pace at which air absorbs heat. Further evidence from the data shows that, at all flow rates, the IWSAH consistently displays a considerably greater CHT than the ISWSAH. This outcome is attributed to the enhanced heat transfer rate and overall functionality of the IWSAH. As a result, when comparing the Inclined Winglet SAH to the Inclined Sinusoidal SAH, the maximum CHT rise is greater than 1.12.

The thermo-hydraulic efficiencies of SAHs are used to evaluate their thermo-hydraulic performance. The thermo-hydraulic efficiency of an SAH measures its capability to transfer thermal energy from the solar collector to the air stream, taking into consideration elements such as pressure drop and fluid dynamics of the system^43,44^. The composite measurement includes both the pressure drop characteristics & heat transfer efficiency of SAH. Figure 16 illustrates the thermo-hydraulic efficiency attained by the two SAH systems during the evaluation. The ISWSAH demonstrated thermo-hydraulic efficiencies of 24.2%, 36.7%, and 48.5% in the experiment, corresponding to flow rates of 0.01, 0.02, and 0.03 kg/s, respectively. However, for the same analyzed stream. The results suggest that, within the range of flow rates examined, the Inclined Winglet design exhibits higher thermo-hydraulic efficiency in comparison to the Inclined Sinusoidal structure.

The findings of the economic and environmental study are displayed in Table 5. The superiority of the IWSAH configuration over the ISWSAH arrangement is visible in all categories. The IWSAH technology offers superior energy production, better energy conversion efficiency, decreased unit cost of energy generation and heightened return on investment.

- IWSAH system achieved an energy payback in a mere 1.3 years, whereas the ISWSAH system required 1.62 years for the same outcome.
- The energy production factor is higher for IWSAH, yielding 2.96, while ISWSAH only provides 2.71.
- The comparative analysis demonstrates that the life cycle conversion efficiency is 43.6% for IWSAH and 39.1% for ISWSAH, which is a noteworthy factor to consider.
- The emissions of other gases, such as sulfur dioxide (SO_2_), nitrogen oxide (NO), and carbon dioxide (CO_2_), are also decreased to a similar extent. In terms of economic considerations, the IWSAH surpasses the ISWSAH in economic analysis.

**Table 5:** Outcome of environmental and economic study.

S. No.	Parameters	ISWSAH	IWSAH
1.	EPBT (Years)	1.62	1.33
2.	EPF	2.71	2.96
3.	LCCE (%)	39.1	43.6
4.	CO_2_ Emission (kg)	2201.3	2147.2
5.	NO Emission (kg)	16.6	16.3
6.	SO_2_ Emission (kg)	6.9	6.7
7.	CO_2_ Mitigation	139.20	125.17
8.	CRF	0.11	0.11
9.	SFF	0.017	0.01
10.	FAC (₹)	3053.9	2938.1
11.	YMC (₹)	305	293
12.	S (₹)	5200	5100
13.	YSC (₹)	90.79	87.29
14.	Annualized Cost (₹)	3268.5	3195.7

## Conclusion

The major objective of this work is to evaluate the performance of an SAH with reconstructed roughness elements. Two distinct winglet combinations will be used in the study: an inclined, triangular combination and an inclined, sinusoidal combination. The following conclusions can be drawn after comparing these two different types of roughness aspects:

- The output air temperature was higher in IWSAH configurations due to improved internal turbulence and a quicker rate of heat transfer than in ISWSAH configurations.
- When compared to the ISWSAH system at flow rates of 0.01, 0.02, and 0.03 kg/s, the IWSAH system shows an increase in average useful power (or) average energy gain of 4.36%, 6.08%, and 6.15% because of the achieved temperature differential and the wider heat transfer range.
- The IWSAH system demonstrates average efficiencies of 29.5%, 56.2%, and 73.1% within the specified flow rate ranges on the observed day. In contrast, the ISWSAH system only attains average efficiencies of 28.2%, 53.1%, and 68.8%. The IWSAH outperforms the ISWSAH in terms of system efficiency, with improvements of 4.6%, 5.8%, and 6.25%.
- In addition, the average thermo-hydraulic efficiency range of the IWSAH system is noticeably wider. The IWSAH system’s average thermo-hydraulic efficiency is 7.43%, 6.81%, and 6.39% higher than the ISWSAH systems for flow rates respectively.
- The convective heat transfer coefficient measured on the study day for the IWSAH system is significantly greater. This range indicates a 1.12-fold augmentation in the convective heat transfer coefficient compared to the ISWSAH system.
- The heat loss via the upper surface of the IWSAH system is decreased by 7.87%, 8.97%, and 9.75% compared to the ISWSAH designs. The main factor contributing to the enhanced thermal efficiency of the IWSAH system is the reduction in heat dissipation.
- The comparative analysis demonstrates that, in terms of energy, economic, and environmental factors, the IWSAH geometry outperforms the ISWSAH geometry for the selected site.

## Data Availability

The data supporting the findings of this study are available from the first author, Vijayakumar Rajendran (email: talk2vijayakumar89@gmail.com), upon reasonable request.
